# The aggregate index of systemic inflammation (AISI): a novel predictor for hypertension

**DOI:** 10.3389/fcvm.2023.1163900

**Published:** 2023-05-17

**Authors:** Jiaming Xiu, Xueqin Lin, Qiansheng Chen, Pei Yu, Jin Lu, Yanfang Yang, Weihua Chen, Kunming Bao, Junjie Wang, Jinlong Zhu, Xiaoying Zhang, Yuxiong Pan, Jiabin Tu, Kaihong Chen, Liling Chen

**Affiliations:** ^1^ Department of Cardiology, Longyan First Affiliated Hospital of Fujian Medical University, Longyan, China; ^2^ The Third Clinical Medical College, Fujian Medical University, Fuzhou, China; ^3^ Department of Cardiology, Fuzhou First Affiliated Hospital of Fujian Medical University, Fuzhou, China

**Keywords:** hypertension, inflammation, aggregate index of systemic inflammation, cardiovascular mortality, NHANES

## Abstract

**Objective:**

Inflammation plays an important role in the pathophysiology of hypertension (HTN). Aggregate index of systemic inflammation (AISI), as a new inflammatory and prognostic marker has emerged recently. Our goal was to determine whether there was a relationship between HTN and AISI.

**Methods:**

We analyzed patients with HTN from the National Health and Nutrition Examination Survey (NHANES) from 1999 to 2018. The primary end point was cardiovascular mortality. A total of 23,765 participants were divided into four groups according to the AISI quartile level. The association between AISI and cardiovascular mortality in patients with HTN was assessed by survival curves and Cox regression analyses based on NHANES recommended weights.

**Results:**

High levels of AISI were significantly associated with cardiovascular mortality in patients with HTN. After full adjustment for confounders, there was no significant difference in the risk of cardiovascular mortality in Q2 and Q3 compared to Q1, while Q4 (HR: 1.91, 95% CI: 1.42–2.58; *P* < 0.001) had a higher risk of cardiovascular mortality compared to Q1. Results remained similar in subgroup analyses stratified by age (*P* for interaction = 0.568), gender (*P* for interaction = 0.059), and obesity (*P* for interaction = 0.289).

**Conclusions:**

In adults with HTN, elevated AISI levels are significantly associated with an increased risk of cardiovascular mortality and may serve as an early warning parameter for poor prognosis.

## Introduction

Hypertension (HTN) is a highly prevalent disease worldwide. From 1990 to 2019, the number of people aged 30–79 years with HTN doubled (1). The prevalence of HTN continues to increase due to factors associated with an aging population and changing lifestyles (2). HTN is a major preventable risk factor for cardiovascular disease (CVD) and death (3, 4), and it continuously damages the heart, brain, kidneys, and other target organs. HTN is usually monitored at the primary health care level or at home by measuring blood pressure (BP) using a sphygmomanometer to guide adjustment of medication regimens. Only a small percentage of patients visit the hospital for annual evaluation, usually focusing on biochemical metabolic markers such as lipids, glucose, and uric acid. The impact of whole blood cells on the prognosis of patients with HTN is often ignored by either clinicians or patients, and more valid blood indicators are lacking to aware the prognosis of patients.

Chronic low-grade inflammation appears to be an early feature of many chronic diseases and is thought to be associated with the development of HTN (5). Biomarkers of inflammation, including C-reactive protein (CRP), various cytokines, and products of the complement pathway, are elevated in HTN (6, 7), suggesting that HTN is in some way an inflammatory disease. As inflammation levels increase, HTN become more susceptible to cardiovascular events such as myocardial infarction (MI) and stroke (8), while the relationship between inflammation on cardiovascular mortality in HTN is unclear.

Aggregate index of systemic inflammation (AISI: neutrophils (NEU) * platelets (PLT) * monocytes (MONO)/lymphocytes (LYM)) is an indicator for the comprehensive assessment of systemic inflammatory state through the whole blood cells, which is an easy accessible metric. AISI is a novel prognostic biomarker that has been focused on patients with idiopathic pulmonary fibrosis (IPF). Studies have shown that it can significantly distinguish patients with IPF from healthy subjects, and AISI level is independently associated with poor prognosis (9, 10). AISI was also found to be significantly associated with poor prognosis in patients with viral pneumonia (11). However, few studies investigate the predicted value of AISI for the outcome of HTN. Therefore, the aim of this study was to explore whether AISI is independently associated with prognosis in HTN.

## Methods

### Study population

The NHANES dataset is a nationally representative cross-sectional survey conducted by the National Center for Health Statistics (NCHS). This is a large-scale ongoing probability survey of representatives of non-hospitalized civilian households in the United States, conducted once a year, every two years for a cycle. This study used 10 cycles of the NHANSE dataset from 1999 to 2018 for retrospective analysis.

**Figure 1:**
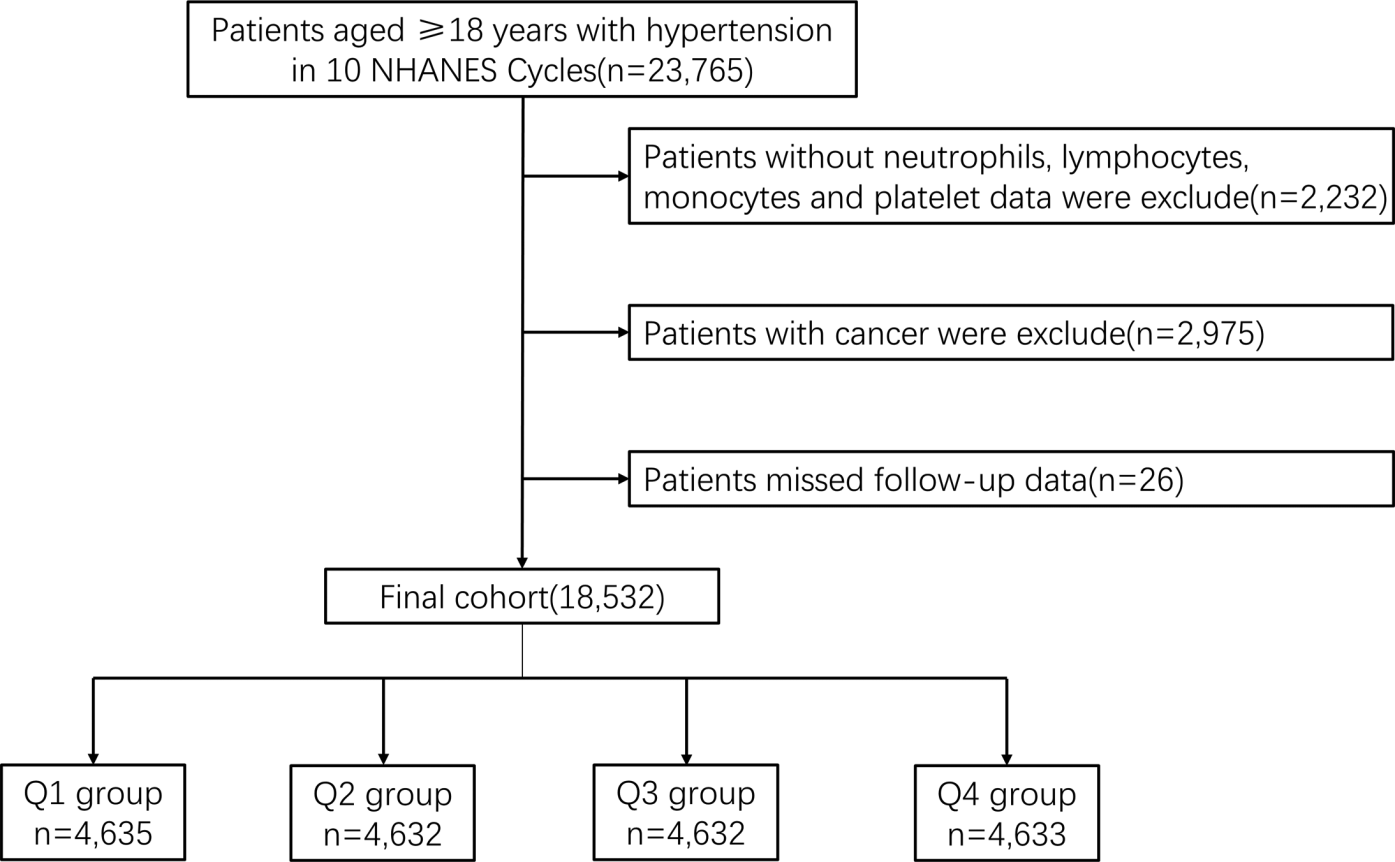
Flowchart of study design.

A total of 23,765 patients diagnosed with HTN over the age of 18 years were included in 10 consecutive NHANES survey cycles from 1999 to 2018. HTN was defined as mean systolic blood pressure (SBP) above 140 mmHg, mean diastolic blood pressure (DBP) above 90 mmHg or current use of prescription medication for HTN. Untreated participants responded to the question “Have you ever been told by a doctor or other health professional that you have high blood pressure (also known as HTN)?” and answered affirmatively to this question were also classified as having HTN. We excluded participants who lacked data on NEU, LYM, MONO and PLT (*n* = 2,232), and who had cancer (*n* = 2,975). In addition, 26 participants who failed follow-up were excluded. In the end, a total of 18,532 participants were enrolled in the retrospective study (Figure 1).

### Study variable

All BP measurements (SBP and DBP) were performed at the Mobile Examination Center (MEC). After resting quietly in a seated position for 5 min and determining the participant's maximum inflation level (MIL), three consecutive BP readings were obtained. If the BP measurement is interrupted or incomplete, a fourth attempt may be made. Participants with either of the following on both arms were excluded from the examination: rash, gauze dressing, cast, edema, paralysis, tubes, open ulcers or wounds, atrophy of the arm, AV shunt, and radical mastectomy. BP measurements were performed on the right arm unless specific conditions prohibited the use of the right arm or if the participant reported any reason why BP measurements should not be performed on the right arm.

Participants self-reported age, gender, race, smoking status, alcohol consumption, and antihypertensive drugs use. Laboratory measurements such as creatinine (Cr), NEU counts, and LYM counts were collected using automated blood analysis equipment. A document on the website of the NCHS provides detailed procedures for obtaining laboratory measurements. Comorbidities and prescriptions were determined by using self-reported health status diagnoses within the past 12 months.

### Primary outcome

The primary outcome was cardiovascular death. Causes of death were classified using the International Classification of Diseases, 10th Revision (ICD-10). Cardiovascular mortality was classified using ICD-10 codes I00-I078. For participants in NHANES 1999–2018, mortality follow-up data was available through December 31, 2019.

### Statistical analysis

We used the weights recommended by the NHANES to calculate the group-specific weights. Continuous variables were expressed as mean ± standard deviation and categorical variables were expressed as counts (percentages). Baseline characteristics were analyzed by ANOVA and chi-square test for continuous and categorical variables respectively.

To assess the relationship of cardiovascular mortality between AISI and HTN, Kaplan-Meier survival analysis curve and Cox regression analyses were performed. Three models were developed for Cox regression: unadjusted model, minimally adjusted model (age, gender) and fully adjusted model (age, gender, race, body mass index (BMI), smoking, drinking, diabetes mellitus (DM), coronary heart disease (CHD), chronic kidney disease (CKD), chronic heart failure (CHF), Cr, drugs, and SBP). In addition, we investigated the relationship of cardiovascular mortality between AISI and different subgroups (age, gender, and obesity).

All data were obtained using R software (version 4.0.4; R Statistical Calculation Foundation, Vienna, Austria), two-sided *P* values < 0.05 indicated significance in all analyses.

## Results

### Baseline characteristics

Among the 18,532 patients enrolled, the mean age was 55.3 ± 0.2 years, and 9,430 (50.9%) were female. Of them, 7,832(42.3%) are Non-Hispanic White. The whole population was divided in quartile categories based on AISI levels: Q1 group (*n* = 4,635), Q2 group (*n* = 4,632), Q3 group (*n* = 4,632), and Q4 group (*n* = 4,633). BMI was higher in the high AISI group (Q1: 30.02 ± 0.13 vs. Q2: 30.59 ± 0.14 vs. Q3: 31.42 ± 0.16 vs. Q4: 31.58 ± 0.17). Patients with higher AISI levels more likely to have commodities including DM (Q1 group: 22.8% vs. Q2 group: 21.2% vs. Q3 group: 24.0% vs. Q4 group: 25.1%), CKD (Q1 group: 20.6% vs. Q2 group: 23.5% vs. Q3 group: 23.3% vs. Q4 group: 29.9%), CHD (Q1 group: 5.6% vs. Q2 group: 6.8% vs. Q3 group: 6.0% vs. Q4 group: 7.5%), CHF (Q1 group: 3.6% vs. Q2 group: 3.5% vs. Q3 group: 4.6% vs. Q4 group: 6.1%), stroke (Q1 group: 5.5% vs. Q2 group: 4.6% vs. Q3 group: 5.0% vs. Q4 group: 7.0%). More patients in high AISI group smoking (Q1 group: 45.1% vs. Q2 group: 46.2% vs. Q3 group: 49.5% vs. Q4 group: 54.5%). Laboratory results showed that as AISI levels increased, patients had elevated white blood cell (WBC), NEU, LYM, Cr, and MONO. Patients were more likely to use antihypertensive drugs in the group with higher AISI levels. There were no significant differences in SBP and DBP at baseline characteristics. More data on baseline characteristics of the study population are detailed in Table 1.

**Table 1:** Baseline characteristics of patients stratified by AISI levels.

Characteristics	Overall (*N* = 18,532)	Q1 (*N* = 4,635)	Q2 (*N* = 4,632)	Q3 (*N* = 4,632)	Q4 (*N* = 4,633)	*P*-value
**Demographic characteristics**						
Age, years	55.3 ± 0.2	55.0 ± 0.4	55.1 ± 0.3	55.1 ± 0.3	55.8 ± 0.4	0.236
Female, *n* (%)	9,430 (50.9)	2,439 (52.0)	2,378 (50.1)	2,348 (51.1)	2,265 (49.2)	0.228
BMI, kg/m^2^	30.96 ± 0.08	30.02 ± 0.13	30.59 ± 0.14	31.42 ± 0.16	31.58 ± 0.17	<0.001
Race, *n* (%)						<0.001
Mexican American	2,943 (15.9)	644 (6.5)	777 (6.3)	814 (6.7)	708 (5.7)	
Non-Hispanic Black	4,837 (26.1)	1,962 (26.6)	1,189 (14.0)	957 (10.8)	729 (7.8)	
Non-Hispanic White	7,832 (42.3)	1,203 (53.0)	1,872 (67.1)	2,175 (72.3)	2,582 (77.1)	
Other Hispanic	1,442 (7.8)	359 (5.4)	391 (5.1)	370 (5.0)	322 (4.1)	
Other Race	1,478 (8.0)	467 (8.5)	403 (7.4)	316 (5.3)	292 (5.2)	
Smoking, *n* (%)	8,961 (48.4)	2,037 (45.1)	2,125 (46.2)	2,283 (49.5)	2,516 (54.5)	<0.001
Drinking, *n* (%)	10,094 (54.5)	2,455 (60.8)	2,556 (63.1)	2,581 (63.2)	2,502 (60.7)	0.326
**Medical history**						
DM, *n* (%)	5,372 (29.0)	1,319 (22.8)	1,300 (21.2)	1,348 (24.0)	1,405 (25.1)	<0.001
CKD, *n* (%)	5,439 (30.2)	1,141 (20.6)	1,243 (23.5)	1,375 (23.3)	1,680 (29.9)	<0.001
CHD, *n* (%)	1,310 (7.1)	264 (5.6)	329 (6.8)	322 (6.0)	395 (7.5)	0.006
CHF, *n* (%)	1,092 (5.9)	211 (3.6)	222 (3.5)	275 (4.6)	384 (6.1)	<0.001
Stroke, *n* (%)	1,269 (6.8)	282 (5.5)	279 (4.6)	295 (5.0)	413 (7.0)	<0.001
**Laboratory examination**						
WBC, 10^9^/l	7.44 ± 0.03	5.83 ± 0.04	6.77 ± 0.03	7.62 ± 0.03	9.13 ± 0.04	<0.001
NEU, 10^9^/l	4.45 ± 0.02	2.91 ± 0.02	3.83 ± 0.02	4.60 ± 0.02	6.07 ± 0.03	<0.001
MONO, 10^9^/l	0.50 ± 0.00	0.44 ± 0.01	0.53 ± 0.00	0.60 ± 0.00	0.74 ± 0.00	<0.001
LYM, 10^9^/l	2.14 ± 0.01	2.26 ± 0.03	2.16 ± 0.02	2.15 ± 0.02	2.03 ± 0.01	<0.001
HBG, g/l	14.29 ± 0.03	14.11 ± 0.04	14.36 ± 0.04	14.36 ± 0.04	14.29 ± 0.04	<0.001
PLT, 10^9^/l	254.20 ± 0.93	212.54 ± 1.29	236.98 ± 1.20	260.67 ± 1.31	295.88 ± 1.78	<0.001
Cr, umol/l	82.85 ± 0.45	81.75 ± 0.64	81.12 ± 0.56	81.47 ± 0.60	86.61 ± 1.10	<0.001
SBP, mmHg	134.26 ± 0.24	134.53 ± 0.41	134.23 ± 0.39	134.16 ± 0.44	134.17 ± 0.39	0.859
DBP, mmHg	74.88 ± 0.21	75.23 ± 0.34	75.50 ± 0.32	74.83 ± 0.30	74.08 ± 0.32	<0.001
**Medicine**						
Antihypertensive drugs, *n* (%)	12,477 (84.1)	3,092 (80.3)	3,091 (79.8)	3,081 (81.8)	3,213 (84.4)	0.001
**Events**						
Cardiovascular death	1,308(7.1)	244(4.5)	296(4.9)	324(5.0)	444(7.3)	<0.001

Values are, *n* (%) or mean ± SD.

BMI, body mass index; DM, diabetes mellitus; CKD, chronic kidney disease; CHD, coronary heart disease; CHF, congestive heart failure; WBC, white blood cell; NEU, neutrophil; MONO, monocyte; LYM, lymphocyte; HGB; hemoglobin; PLT, platelet; Cr, creatinine; SBP, systolic blood pressure; DBP, diastolic blood pressure.

**Table 2:** Cox regression analysis of the associations between AISI and cardiovascular mortality.

	Model1			Model2			Model3		
	HR	95%CI	*P*-value	HR	95%CI	*P*-value	HR	95%CI	*P*-value
**Cardiovascular mortality**									
Group1	ref			ref			ref		
Group2	1.02	0.82–1.26	0.878	1.03	0.85–1.25	0.761	1.12	0.88–1.43	0.362
Group3	1.04	0.80–1.34	0.770	1.09	0.85–1.39	0.501	1.23	0.89–1.69	0.205
Group4	1.68	1.34–2.11	<0.001	1.75	1.39–2.19	<0.001	1.91	1.42–2.58	<0.001

Model 1: No adjusted.

Model 2: Adjusted by age, gender.

Model 3: Adjusted by age, gender, race,BMI, smoking, drinking, DM, CHD, CKD, CHF, Cr, drug, SBP.

BMI, body mass index; DM, diabetes mellitus; CHD, coronary heart disease; CKD, chronic kidney disease; CHF, congestive heart failure; Cr, creatinine; SBP, systolic blood pressure.

### AISI and cardiovascular mortality

**Figure 2:**
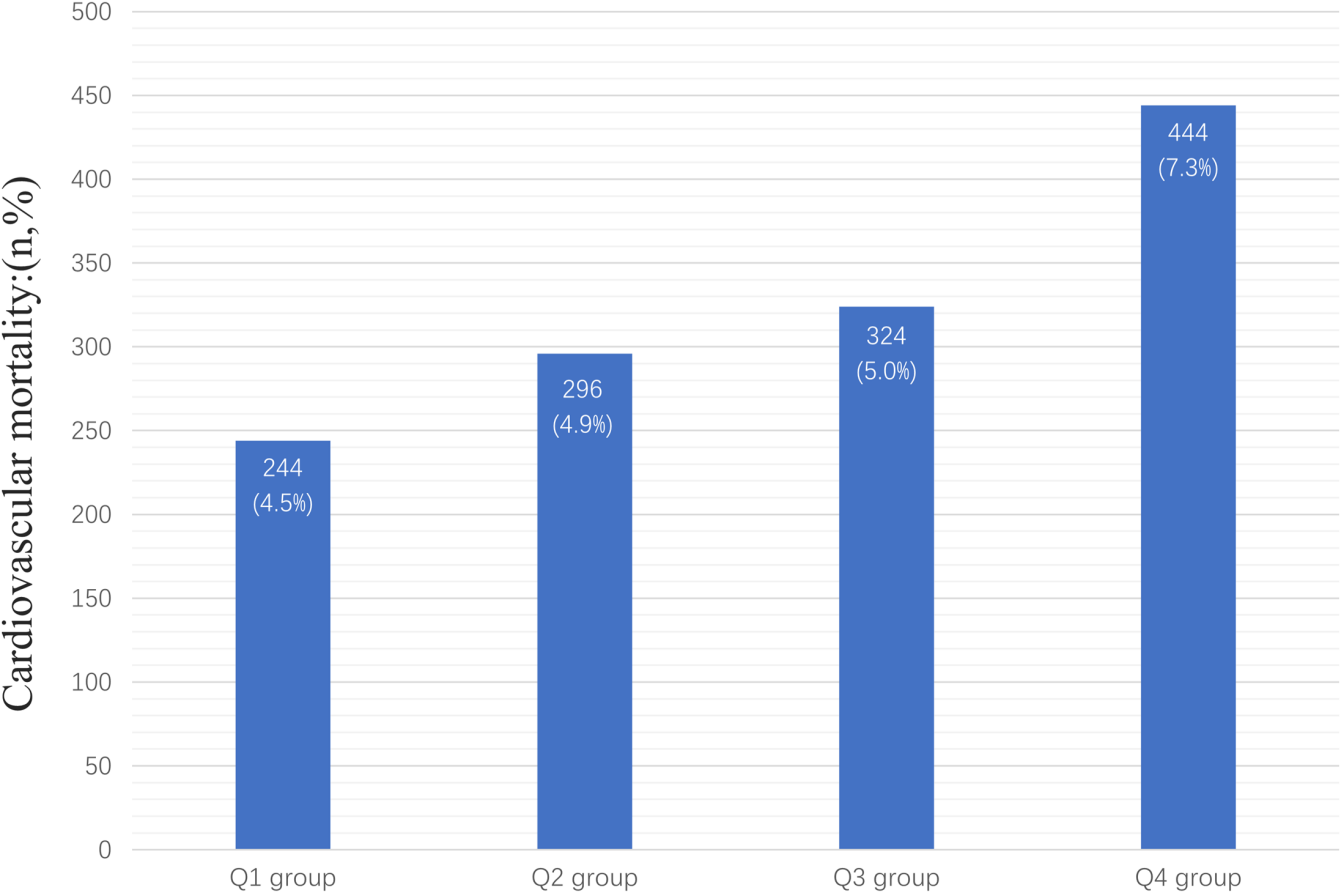
Number and percentage of deaths in each group.

**Figure 3:**
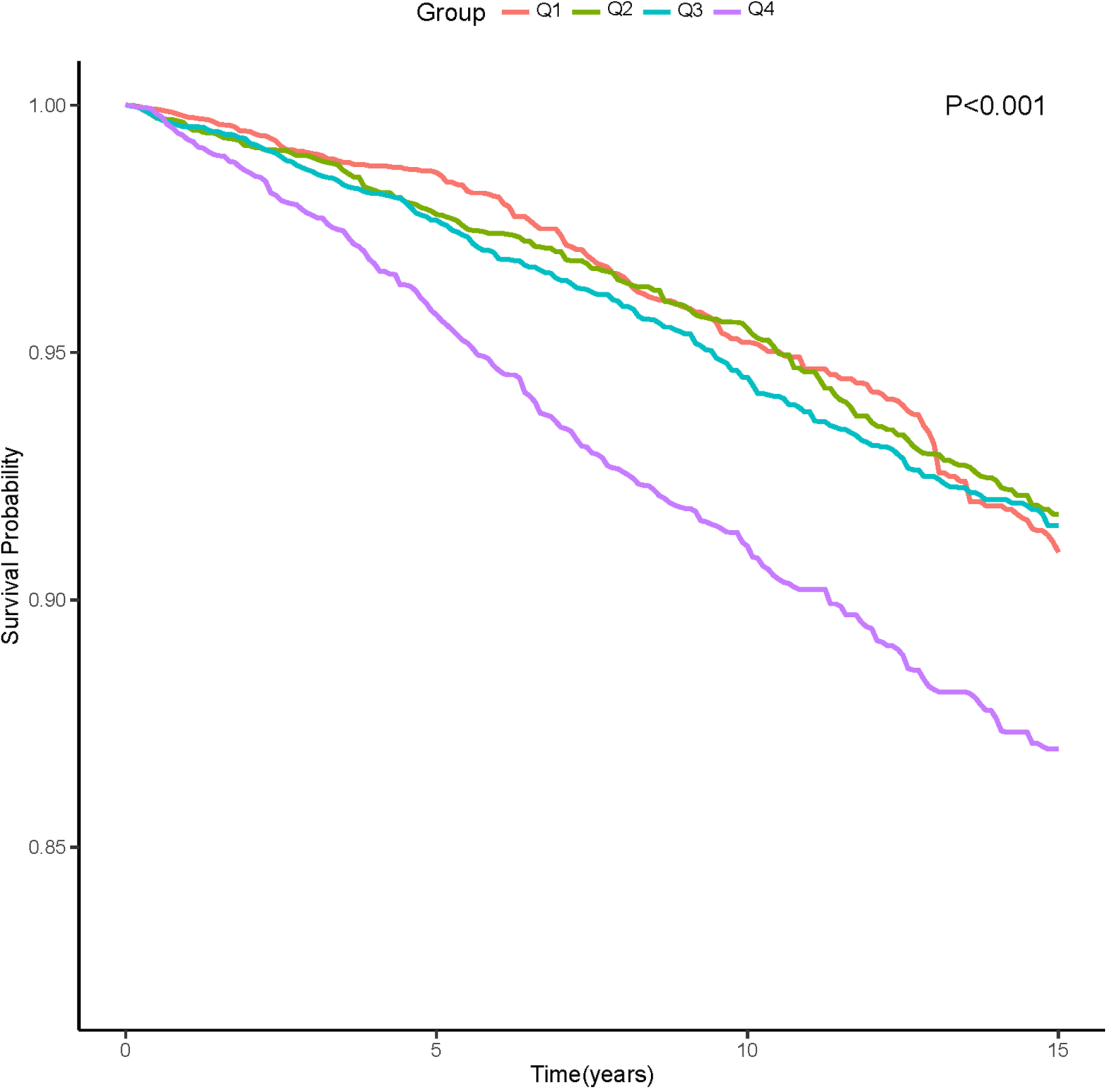
Kaplan-Meier Survival Estimates for long-term cardiovascular mortality (weighted).

In the entire study population, there were 1,308 cardiovascular deaths (7.1%), including 244 deaths in the Q1 group (4.5%), 296 deaths in the Q2 group (4.9%), 324 deaths in the Q3 group (5.0%), and 444 deaths in the Q4 group (7.3%) (Figure 2). We observed that patients with higher AISI levels had higher cardiovascular mortality. Kaplan-Meier survival analysis curves showed that the survival rate was lower in the group with higher AISI (*P*-log rank < 0.001, Figure 3). Univariate Cox regression analysis showed that there was no significant difference in the risk of cardiovascular mortality between Q2 group (HR: 1.02, 95%CI: 0.82–1.26; *P* = 0.878) and Q3 group (HR: 1.04, 95%CI: 0.80–1.34; *P* = 0.770) compared with Q1 group, while Q4 group (HR: 1.68, 95%CI: 1.34–2.11; *P* < 0.001) had a higher risk of cardiovascular mortality compared with Q1 group. In fully adjusted models that included confounding factors such as age, gender, race, BMI, smoking, drinking, DM, CHD, CKD, CHF, Cr, drug, SBP, the Q4 group still had a higher risk of cardiovascular mortality (HR: 1.91, 95% CI: 1.42–2.58; *P* < 0.001) (Table 2).

### Subgroup analysis

**Figure 4:**
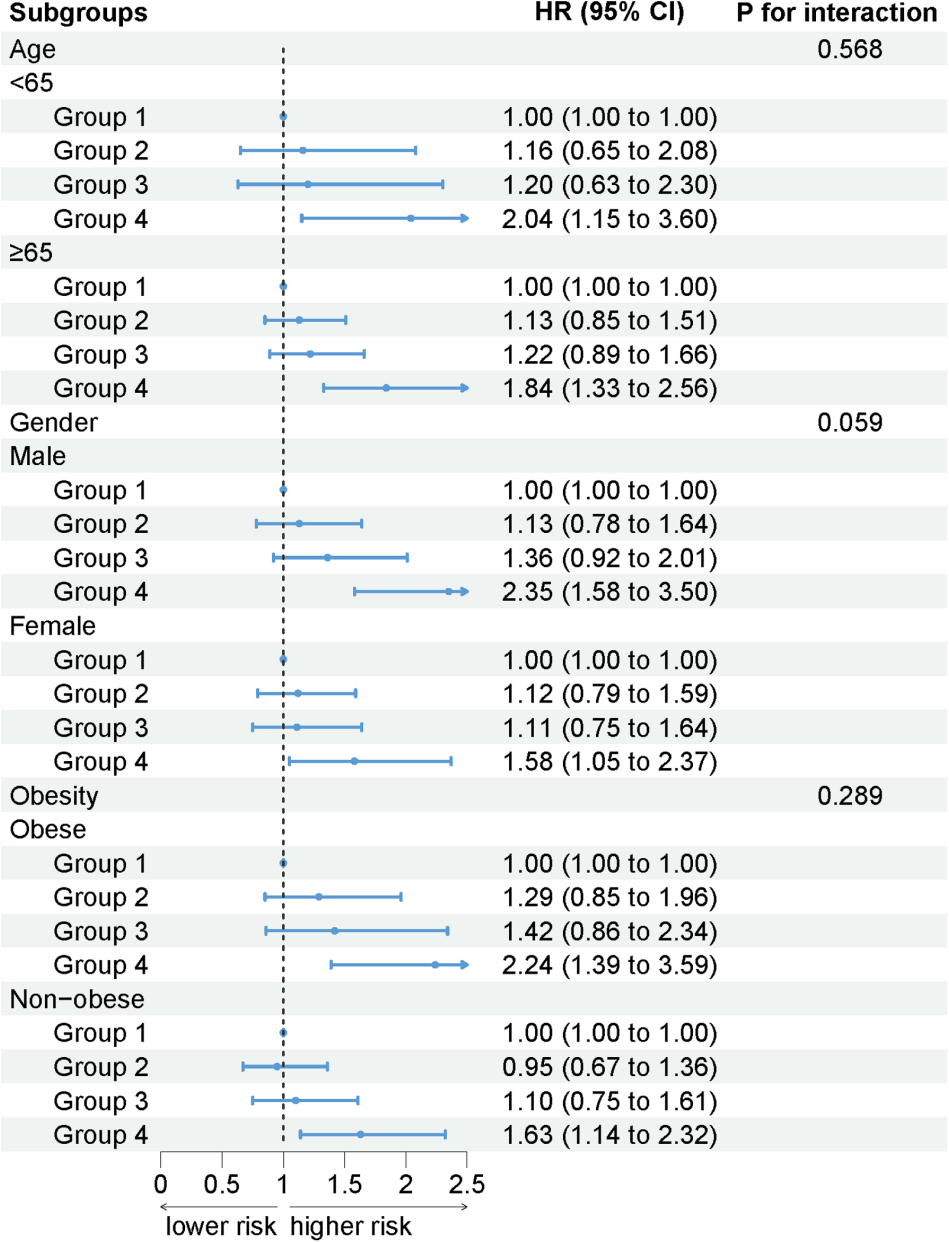
The association between AISI and cardiovascular mortality by selected subgroups (weighted).

The association between AISI and cardiovascular mortality remained unchanged when participants were stratified by age (interaction *P* = 0.568), gender (interaction *P* = 0.059), and obesity (interaction *P* = 0.289). As AISI increased, the risk of cardiovascular mortality increased (Figure 4).

## Discussion

To our knowledge, this study is the first to use the inflammatory marker AISI as a predictor of cardiovascular mortality in patients with HTN. The results showed that the risk of cardiovascular mortality was nearly 1-fold higher in HTN with extremely high AISI, even after full adjustment for confounders. The results remained stable after stratification for age, gender, and obesity.

The impact of inflammation on the prognosis of HTN has received some attention. In the study by Aydin C et al., systemic immune inflammatory index (SII) levels were used to predict stroke in patients with HTN, and SII levels increased the risk of stroke (12). This suggests an association between inflammation and adverse outcomes of HTN, our study focus on cardiovascular mortality in HTN, where is another serious adverse outcome. Akyuz A et al. found that SII was an independent predictor of non-dipper HTN and that high SII levels would increase the risk of all-cause mortality in patients with non-dipper HTN (13). Our study population included all eligible patients with HTN and facilitated generalization in the population. In addition, SII is calculated from NEU, PLT, and LYM counts, whereas AISI also includes MONO counts that are responsive to inflammation, and this index may provide a more comprehensive picture of systemic inflammation. In a study of patients with IPF, Zinellu A et al. found that only AISI was significantly associated with poor prognosis, and it had better predictive value compared to related inflammatory indicators such as neutrophil-to-lymphocyte ratio (NLR), platelet-to-lymphocyte ratio (PLR), SII and systemic inflammation response index (SIRI) (9).

HTN is a low-grade inflammatory state accompanied by elevated cytokine levels. AISI predicts that the risk of cardiovascular death from HTN may be related to the inflammatory effects of its individual inflammatory cells. In the innate immune system, NEU and MONO can raise BP through their actions in the blood vessels and kidneys (14, 15). In the adaptive immune system, activated B cell and T cell have been shown to raise BP (16, 17). Inflammation not only leads to increased BP but also plays a central role in end-organ damage in HTN (18). A relatively new mechanism suggests that one of the major functions of activated NEU is to expel their DNA as a network of chromatin structures called neutrophil ectodomains (NETs). NETs are released into the extracellular space along with other granule proteins, including neutrophil elastase (NE), myeloperoxidase (MPO), and caseinase G. This excretion of DNA generates a pro-oxidative, pro-inflammatory and pro-thrombotic environment that can induce endothelial damage (19), which is also associated with atherosclerosis, coronary artery disease (CAD), endotoxic shock and target organ damage (20–22), leading to cardiovascular events. MONO are involved in vascular accumulation, upregulation of IL-1β, IL-6 and TNF-α expression in the brain, formation of reactive oxygen species (ROS), promotion of angiotensin-II (AngII) and other mechanisms that form atherosclerosis, brain injury, heart and kidney injury (23). Activated T cell infiltrate tissues and produce cytokines, including interleukin-17A, promoting renal and vascular dysfunction leading to end-organ injury (17, 24). An increase in adenosine diphosphate-induced PLT aggregation has been observed in patients with HTN (25). This activation is due to impaired uptake of L-arginine and PLT nitric oxide production in HTN (26). High PLT counts are associated with CVD including CAD and stroke, increasing cardiovascular mortality (27–29). For example, in patients with atherosclerosis, activated PLT and endothelial cells after subendothelial matrix exposure recruit the aforementioned NEU and MONO, which promote thrombosis through tissue factor (TF) delivery and the formation of NETs (30, 31). In conclusion, inflammation can promote vascular remodeling, leading to vascular fibrosis and CVD (32), as well as act on end organs in HTN, leading to increased mortality.

Our study shows that AISI reflects the level of inflammation in HTN and is associated with cardiovascular mortality. In clinical practice, a simple combination of whole blood cells is suited to detecting patients with high levels of inflammation, thus enabling clinicians to rank inflammatory factors and intervene. If localized infectious inflammation is present, the appropriate anti-microbial therapy needs to be selected according to the pathogen. Whereas HTN is a chronic state of low-grade inflammation, this inflammation is an aseptic (non-infectious) inflammation, unlike acute inflammation due to local injury. Antibiotics are generally not recommended for HTN, but when selecting antihypertensive drugs, drugs with certain anti-inflammatory properties, such as angiotensin-converting enzyme inhibitors (ACEI) and angiotensin receptor blocker (ARB), are available and have the best anti-inflammatory activity (33). In addition, a relatively new drug to improve heart failure (HF) is known as Sacubitril/Valsartan (S/V), which has some anti-inflammatory effects while lowering BP (34, 35), and is particularly suitable for patients with HTN combined HF. Patients with HTN combined with diabetes can use SGLT-2 to lower blood glucose with some antihypertensive and anti-inflammatory effects (36, 37). In addition, our study is the first to apply this index to the cardiovascular system, which is suggestive for future studies and will help to conduct multicenter prospective studies and randomized controlled trials to further confirm our hypothesis.

Our study has some limitations. Our study is a retrospective analysis of NHANES and there may be confounding factors, so we performed cox regression, and next we plan to do further cohort studies or randomized controlled trials. Second, a single blood draw is not a good proxy for a patient's physical status, which may change considerably during long-term follow-up. This may change with long-term follow-up.

## Conclusion

AISI is an index to assess the inflammatory status of patients with HTN. In the adult population, elevated AISI levels are significantly associated with an increased risk of cardiovascular mortality due to HTN. High AISI values in patients with HTN can be used as an early warning parameter for poor prognosis. These findings could contribute to the development of new treatments to control low-grade inflammation and hypertensive damage. However, further randomized clinical trials are needed to confirm this idea.

## Data Availability

Publicly available datasets were analyzed in this study. This data can be found here: https://www.cdc.gov/nchs/nhanes/index.htm.
